# In search of the locus coeruleus: guidelines for identifying anatomical boundaries and electrophysiological properties of the blue spot in mice, fish, finches, and beyond

**DOI:** 10.1152/jn.00193.2023

**Published:** 2024-06-06

**Authors:** Amelien Vreven, Gary Aston-Jones, Anthony E. Pickering, Gina R. Poe, Barry Waterhouse, Nelson K. Totah

**Affiliations:** ^1^Helsinki Institute of Life Science (HiLIFE), University of Helsinki, Helsinki, Finland; ^2^Faculty of Pharmacy, University of Helsinki, Helsinki, Finland; ^3^Neuroscience Center, University of Helsinki, Helsinki, Finland; ^4^Brain Health Institute, Rutgers University, Piscataway, New Jersey, United States; ^5^Anaesthesia, Pain & Critical Care Sciences, School of Physiology, Pharmacology & Neuroscience, University of Bristol, Bristol, United Kingdom; ^6^Department of Integrative Biology and Physiology, University of California, Los Angeles, California, United States; ^7^Department of Psychiatry and Biobehavioral Sciences, University of California, Los Angeles, California, United States; ^8^Department of Neurobiology, University of California, Los Angeles, California, United States; ^9^Department of Cell Biology and Neuroscience, Rowan University School of Osteopathic Medicine, Stratford, New Jersey, United States

**Keywords:** anatomy, brainstem, calcium imaging, locus coeruleus, single-unit recording

## Abstract

Our understanding of human brain function can be greatly aided by studying analogous brain structures in other organisms. One brain structure with neurochemical and anatomical homology throughout vertebrate species is the locus coeruleus (LC), a small collection of norepinephrine (NE)-containing neurons in the brainstem that project throughout the central nervous system. The LC is involved in nearly every aspect of brain function, including arousal and learning, which has been extensively examined in rats and nonhuman primates using single-unit recordings. Recent work has expanded into putative LC single-unit electrophysiological recordings in a nonmodel species, the zebra finch. Given the importance of correctly identifying analogous structures as research efforts expand to other vertebrates, we suggest adoption of consensus anatomical and electrophysiological guidelines for identifying LC neurons across species when evaluating brainstem single-unit spiking or calcium imaging. Such consensus criteria will allow for confident cross-species understanding of the roles of the LC in brain function and behavior.

## Graphical abstract

**Figure:**
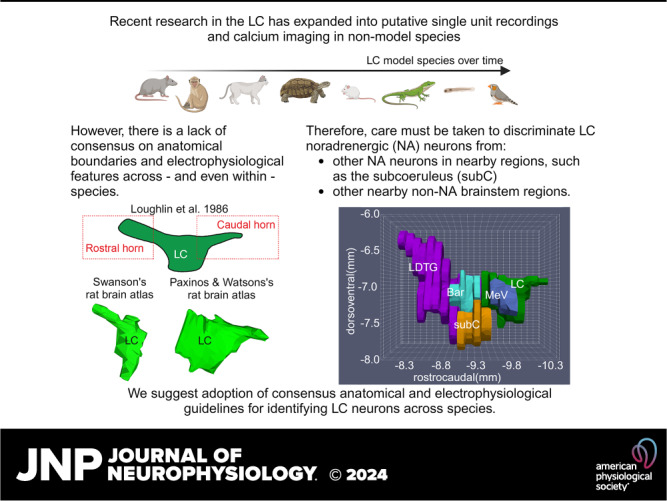

## INTRODUCTION

Our understanding of human brain function has been greatly enhanced through insights gained from the brains of organisms with analogous structures. One brain structure with neurochemical and neuroanatomical homology throughout vertebrate species is the nucleus locus coeruleus (LC). The LC contains ∼14 neurons in the zebrafish and ∼70,000 in the human brain and provides the forebrain’s primary source of norepinephrine (NE) (1, 2). The diminutive size of the LC belies its involvement in nearly every aspect of brain function. The LC projects to the spinal cord, brainstem, midbrain, cerebellum, and most areas of the forebrain, lending credence to the idea of a global connectome that is homologous across species (3–5). However, the question of whether the LC-NE system serves a generalizable role across all species remains unanswered.

One of the most studied functions of the LC-NE system is its contribution to learning and memory, which has been extensively examined in rats and nonhuman primates using single-unit recordings before, during, and after learning (6–8). Bird song learning is another model for studying the involvement of the LC-NE system in learning. Avian neuronal circuits involved in learning bird song are well-defined and several lines of evidence implicate NE in bird song acquisition (4). What has been lacking, however, are recordings of activity in the avian LC during learning or behavior of any sort.

Recently, Katic et al. (9) performed, to our knowledge, the first putative LC single-unit electrophysiological recordings in the avian songbird, the zebra finch. Their work shows that song learning in a social context (via live singing of conspecifics), as opposed to artificial song playback, specifically activates avian hindbrain neurons that may be LC-NE neurons (9). However, the putative LC-NE neurons recorded in the zebra finch did not match the classical definition of LC-NE neurons derived from rats and nonhuman primates (i.e., a 2–3 ms duration of the entire waveform, mean spontaneous firing rate of ∼1 Hz, and a biphasic response to a brief sensory stimulus). This could imply that either the cells recorded in the avian brainstem were not LC-NE neurons, or that the avian LC may be physiologically different from mammalian LC. Thus, this study highlights a challenging conundrum in our field, which is the lack of knowledge about the cell type(s), local neurotransmitters, and physiology of LC neurons in most species relative to the few commonly studied mammalian species. Until we discover what features of the LC are shared across species, some caution is warranted when interpreting purported LC neuronal activity in species emerging as new models for studying the LC.

Here, we seek to provide some guidelines that would allow researchers working with different species—from the classical (i.e., mice and rats) to the exotic (i.e., fish, finches, and beyond)—to target the LC with confidence and make a direct link between their recordings of single-neuron activity and an understanding of the role of the LC in behavior and brain function across species.

### The Four Key Electrophysiological Criteria for Identifying LC-NE Neurons Were Derived from Rodents, Cats, and Nonhuman Primates

**Figure 1.**
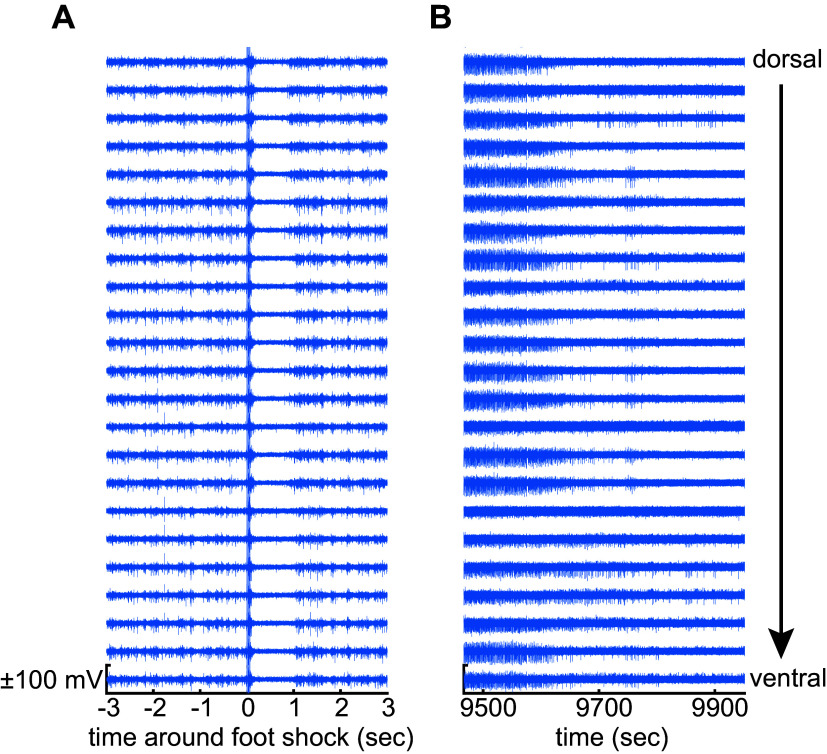
Feedback inhibition distinguishes locus coeruleus (LC) neurons from non-LC neurons. *A*: the highpass (500 Hz) filtered signal recorded on a 23-electrode linear array in a ±3-s window around a 5.0 mA foot shock in the urethane-anesthetized rat. Increased spiking after the stimulus releases norepinephrine (NE) locally, which causes feedback inhibition via NE binding to the a2-adrenergic receptor. Data from one example recording (*N* = 1). *B*: spiking on the same electrodes after clonidine injection (50 µg/kg ip). Clonidine was injected at the start of the recording trace. Data from one example recording (*N* = 1).

**Figure 2.**
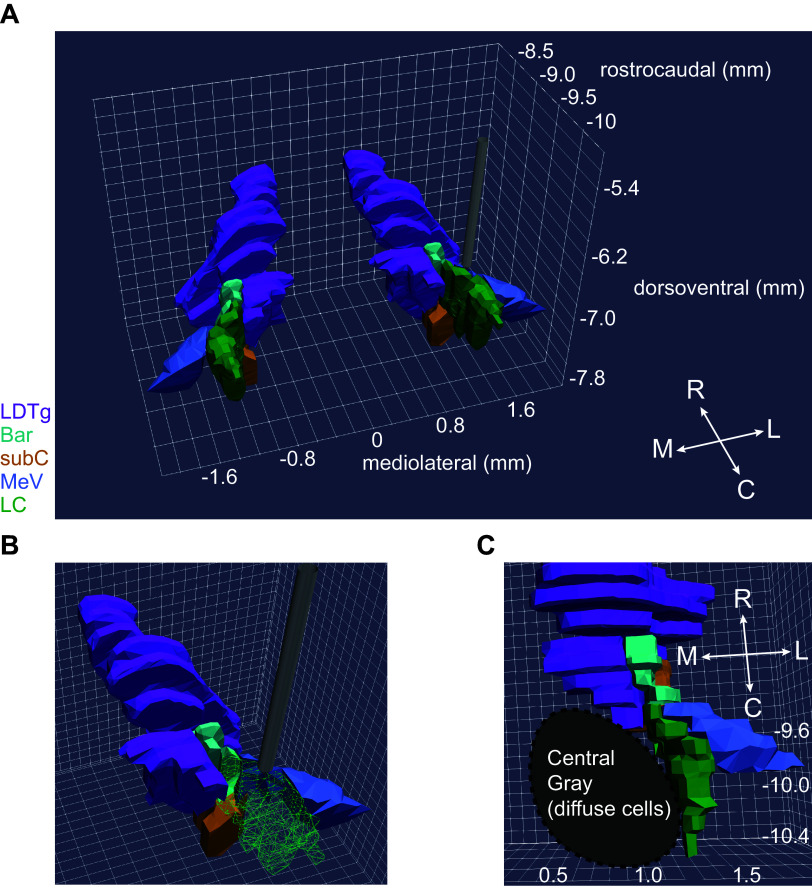
The depth of the locus coeruleus (LC) and its close proximity to multiple brainstem nuclei pose a challenge to accurately targeting the LC, which necessitates the use of electrophysiological features that distinguish LC neurons. *A*: a 100-µm electrode (gray) is shown rostral to the LC (green) and surrounding structures. Axis values are distance in mm from Bregma in the rat brain. *B*: a wire frame rendering of the LC (green) exposes the regions closely surrounding the LC. *C*: a ventral view of the brainstem showing convergence of multiple non-noradrenergic brainstem nuclei at the rostral end of the LC. The laterodorsal tegmental nucleus (LDTg, purple), Barrington’s nucleus (Bar, cyan), and the mesencephalic trigeminal nucleus (MeV, blue) squeeze the rostral LC, whereas the noradrenergic cells of the subcoeruleus (SubC, orange) sit ventral to the rostral LC. Immediately medial to the LC are diffuse non-noradrenergic cells of the pontine Central Gray. In all panels, the three-dimensional (3-D) images were rendered from the two-dimensional (2-D) coronal sections in Paxinos & Waston’s Rat Brain Atlas using the 3-D Brain Atlas Reconstructor service (37, 38).

The mammalian LC has been intensively investigated over the past 50 years, but in a limited number of species (primarily rodents, cats, and nonhuman primates). The physiological attributes of putative LC neurons in other species should be considered and contrasted against what we know from the heritage of LC recordings in the more common laboratory mammalian species. In rats and nonhuman primates, the LC is identifiable as a structure containing almost entirely NE neurons that are densely packed into a small volume (10, 11). Aston-Jones and Bloom (12, 13) and Foote et al. (14) provided the first functional descriptions of the properties of these NE cells in behaving rats and nonhuman primates (12, 14). Cells with those properties were subsequently found in other species (e.g., cat, rabbit, and guinea pig) and are presumed to be LC-NE neurons in those species, but with the important difference that they are interdigitated with non-NE neurons in the LC of those species. In mice, rats, and nonhuman primates, LC-NE neuron activity is characterized by four distinguishing features. First, the duration of the extracellular action potential is relatively long (2 to 3 ms for the duration of the entire waveform), especially compared with other nonaminergic and noncholinergic forebrain neurons. Second, the spontaneous firing rate of LC-NE neurons is, on average across the recorded population, around 1 Hz in anesthetized, sleeping, waking, and cognitive task-engaged preparations (12, 14–18). The population mean firing rate is not higher than 2 Hz. However, the range of firing rates of individually recorded neurons can vary from 0.5 to 5 Hz in the anesthetized and the cognitive task-engaged preparations (19, 20). Still, there have been reports of individual LC neurons discharging up to ∼15 Hz in awake rats under stress conditions in a foot shock fear conditioning paradigm (8). An ∼15 Hz firing rate matches the maximal capacity for reliable conduction of action potentials by noradrenergic fibers, which are exclusively thin and unmyelinated (21). In specific, transient, state-dependent conditions, the mean spontaneous firing rate of the population can vary: from total quiescence during rapid eye movement (REM) sleep (12) and transient silences during non-REM sleep (12, 22) and urethane anesthesia (23), or elevated to 2 to 3 Hz under conditions of stress (24, 25). Third, LC-NE neurons respond to brief, salient stimuli with a characteristic stimulus-evoked excitation followed by feedback inhibition that produces a biphasic response (26). In anesthetized preparations, noxious stimuli such as a 5 mA foot shock in rats (23) will evoke a biphasic stimulus response in the LC predominantly contralateral to the stimulus; in awake preparations, non-noxious stimuli (e.g., visual, auditory, etc.) can be used to evoke this response (13, 14, 26). Figure 1*A* shows the highpass filtered signal recorded simultaneously across 23 electrodes (25 μm spacing) in a linear arrangement from dorsal to ventral and spanning most of the dorsoventral extent of the rat LC. The biphasic response can be observed across all electrodes as a large-scale neuronal population spiking event (20). This finding matches earlier work showing a stimulus-evoked deflection of the field potential (13). The field potential is the temporal and spatial average field produced by the superposition of transmembrane currents near the electrode (27). The field potential deflection observed by Aston-Jones and Bloom (12, 13) is likely generated by synchronous spiking of many (but not necessarily all) LC neurons (20), which would require the temporally synchronous onset of transmembrane currents across many neurons that are closely packed in space. Finally, the LC activity in these relatively well-studied mammals is also temporarily silenced by the α-2 adrenergic receptor agonist clonidine. An example of this robust silencing effect of clonidine is shown in Fig. 1*B*. The spiking activity (same electrodes as Fig. 1*A*) is diminished over time and appears as a run-down in population spiking. In urethane-anesthetized rats, a 50 µg/kg ip dose of clonidine will inhibit LC neurons completely such that even low amplitude multiunit LC activity cannot be detected (20). Indirect measures of LC activity (e.g., the amount of norepinephrine released by the LC in proportion to its neuronal activity level) suggest that 50 µg/kg ip clonidine inhibits the LC in awake rats, but 10 µg/kg ip does not (28, 29). In the rhesus monkey, a dose of 20 µg/kg clonidine intramuscularly nearly completely suppresses LC activity (30, 31). All of these features (i.e., 2 to 3 ms waveform duration, ∼1 Hz firing rate, biphasic stimulus-evoked response, and clonidine-evoked inhibition) should be considered across mammalian species to distinguish LC-NE neuron activity from cellular activity in adjacent brainstem nuclei, many of which have higher firing rates (e.g., 30–100 Hz) and lack the stimulus-evoked biphasic responses (32–36). In addition to these features of LC neuronal activity, histological verification of the electrode tract and recording site is a necessity because the LC is in close proximity to other cell groups (Fig. 2).

### Advances, and Caveats, in Recording LC-NE Activity in Nonmammals

In nonmammals (e.g., teleost, reptilian, and avian species), the physiological characteristics of LC cells are largely unknown, often because recordings have seldom been attempted. There appear to be only a handful of LC neuron recordings in reptiles, two in vitro recordings for which responses to sensory stimuli were not assessed (39, 40), and one in vivo recording that was recorded in the brainstem and may or may not have included neurons in the LC (41). A recent whole cell in vivo recording of LC-NE neuronal action potentials in the teleost, zebrafish, revealed that spontaneous firing rates were similar to LC-NE neuron recordings in mammals (42). However, testing for a biphasic response to salient stimuli or clonidine-evoked inhibition was not performed. The first in vivo action potential recordings putatively from the LC of an awake, behaving avian species, the zebra finch, reported single-unit recordings that did not match the criteria for identifying LC-NE neurons in rodents, cats, and nonhuman primates. The study reports a “fast spiking” group of seven single units with 30–100 Hz firing rate and a “regular spiking” group of nine single units with an ∼5 Hz mean firing rate (9). Neither group of single units exhibited a biphasic response to the stimulus and the waveform durations (peak to valley latency) were maximally ∼500 µs. In rats, two types of regular spiking single units have been reported, both of which have a slow (population mean ∼1 Hz) firing rate under urethane anesthesia and a biphasic response to noxious foot shock stimuli (5.0 mA, 0.5 ms duration to the contralateral paw). One group of rat LC neurons has narrow action potentials (mean = 460 µs trough-to-afterhyperpolarization latency), but most units (85% of 234 units) have wide action potentials (mean = 1,080 µs trough-to-afterhyperpolarization latency) (20). Similar findings have been recently reported for optogenetically tagged dopamine β-hydroxylase (DBH)-positive LC neurons in mice although specific waveform durations were not reported (43) (Note that the classical definition of LC-NE neurons used the entire duration of the waveform, yielding values of 2–3 ms, whereas recent work has used the peak-trough latency). Using these rodent benchmarks, it would seem that the higher discharge rates, lack of biphasic response to stimuli, and short waveforms reported in the zebra finch are similar to cellular profiles found in adjacent brainstem nuclei of rodents (32, 34, 36). This could imply that either the cells recorded in the avian brainstem were not LC-NE neurons, or that the avian LC may be physiologically different from mammalian LC. Both possibilities could be explored with further study.

Rapid advances in calcium imaging are also increasing the use of larval zebrafish to study LC-NE activity. The transparency of the larval zebrafish permits LC-NE neurons to be directly visualized through a confocal microscope via genetically engineered fluorescent markers of LC-NE neurons. Most zebrafish studies of LC-NE neurons used calcium (GCaMP) imaging, which indirectly infers ongoing activity and therefore does not provide a firing rate for direct comparison to action potential recordings in mammals. Moreover, GCaMP registers activity over a window that is 10^2^ or 10^3^ longer than an action potential (at least for the deployed methods such as GCaMP6), which prevents measurement of the stimulus-evoked biphasic response of LC neurons because the excitatory phase of the biphasic response (when it is convolved with the GCaMP indicator) occludes the postexcitatory suppression of firing (44–46). However, because the cells are visualized and the expression pattern can be genetically restricted to catecholaminergic cells, GCaMP can be used to unequivocally identify single LC-NE neurons and track the relationship of LC-NE activity with slow fluctuations in arousal and presentation of salient stimuli, such as has been done in teleosts (47, 48). Similar GCaMP expression has been used successfully in rodents to identify activity patterns in single LC-NE neurons in vitro (49), in vivo (50), or to measure the bulk activity of many LC-NE neurons in vivo using fiber photometry (45, 51). The use of retrograde expression of GCaMP may allow the activity patterns of LC-NE modules with different axonal projection patterns to be compared (49). However, GCaMP appears to be too slow to observe the spontaneous activation of LC ensembles, which are transient and occur on a scale of less than 100 ms in the rat (52). Because of the long temporal integration by GCaMP and slow fluctuations in LC-NE firing rate, caution is warranted when drawing conclusions about the degree of synchronous activity across LC-NE neurons based on GCaMP in mammalian and nonmammalian species alike.

Overall, it may not be appropriate to extrapolate from rodent LC physiology to determine whether the LC is successfully recorded in other species, such as reptiles, avians, and fish. Differences reported in activity between the avian LC and the mammalian LC may be due to ethological, cytoarchitectonic, or cell type differences between avian and rodent behaviors. For instance, differences exist in LC cytoarchitectonic organization even among mammalian species. In rats, mice, and primates (including humans), the majority of LC neurons are densely packed into a so-called “core” (11, 53, 54). In contrast, the LC-NE neurons in many other mammals are scattered diffusely throughout the dorsolateral pons (55), which makes cross-species comparisons challenging. Moreover, in the cat, diffusely distributed NE neurons are intermingled with GABAergic interneurons and even serotonergic neurons (56–59). It is conceivable that other animals, such as birds, share the feline LC organization: a diffuse and intermingled arrangement of neurochemically diverse cells. Although prior work has shown a LC core of densely packed noradrenergic neurons in the zebra finch dorsal pons that resembles that of many mammalian species (60), there is evidence for intermingling of serotoninergic and cholinergic neurons in the avian LC (61–63). Although the lack of a biphasic response to stimuli by putative LC neurons in the zebra finch may indicate that the avian LC is electrophysiologically distinct from the other mammals that have been recorded, there is also evidence that postexcitation autoinhibition in the avian LC may not be entirely different from the rodent. For instance, recent work has shown that zebra finch LC-NE neurons contain mRNA for α2 noradrenergic receptors (3) suggesting that an autoinhibitory feedback loop, which contributes to the inhibitory component of the biphasic response to stimuli in rodents (26, 64, 65) may be active in the avian LC. Thus, avian LC neurons may be capable of expressing a biphasic response—a possibility that should be further investigated. If α2 adrenergic receptors are indeed universal in LC neurons across species, recordings targeting the LC should seek to include tests of the silencing effects of the α2 noradrenergic receptor agonist, clonidine, to confirm that the neurons recorded are indeed LC-NE neurons. In addition, histological confirmation of recording site is critical given the LC may either be a compact core or diffuse scattering of NE neurons in different species.

### Resolving Quandaries concerning the Anatomical Boundaries and Cell Types of the LC across Species

**Figure 3.**
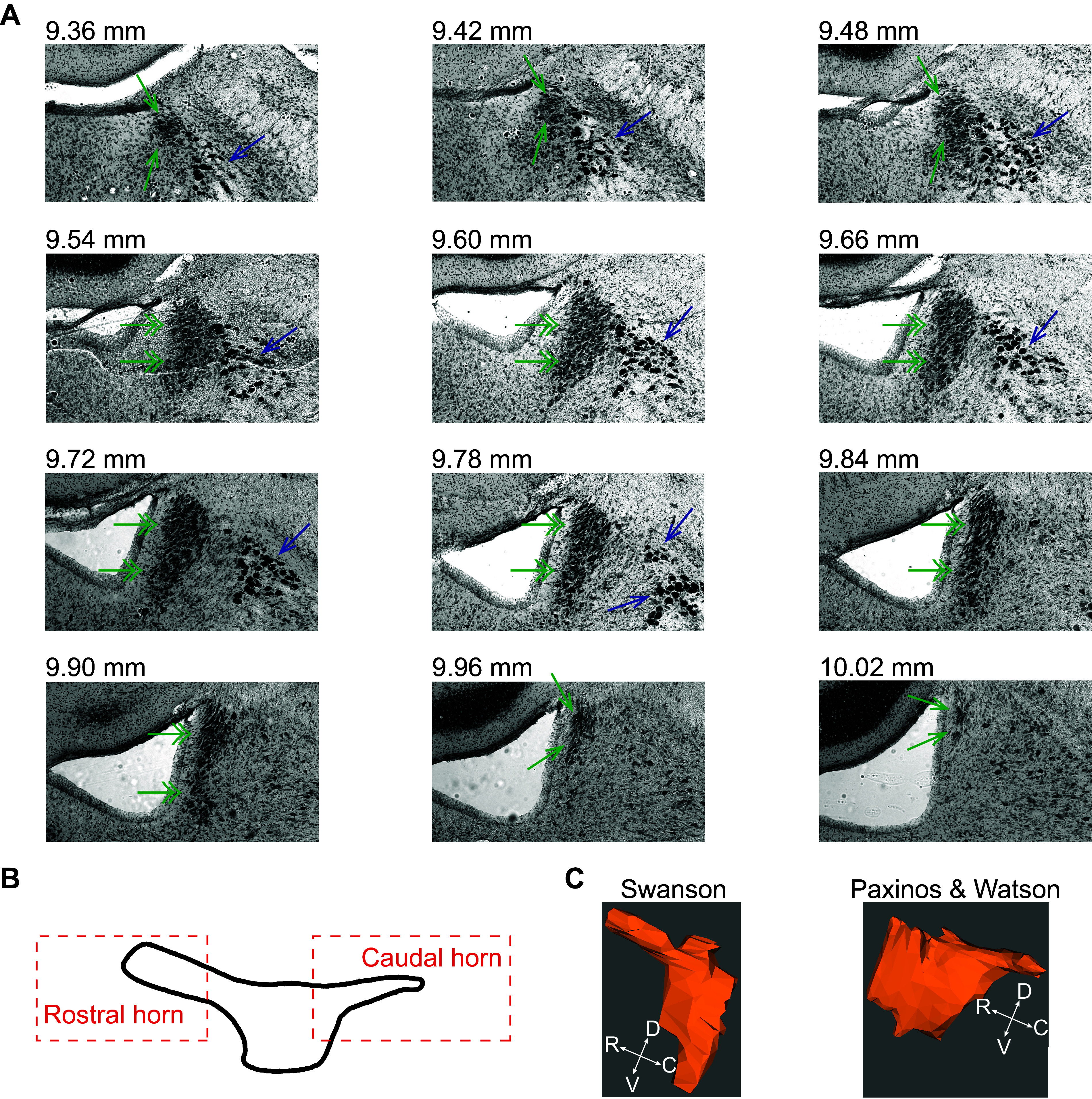
The borders of the locus coeruleus (LC) central core and the rostral and caudal horns are cytoarchitectonically defined using a Nissl stain. *A*: the dense packing of LC neurons is shown from most rostral (*top left*) to most caudal (*bottom right*) in Nissl-stained frontal sections from the rat brain. The image is shown in grayscale. The numbers indicate rostrocaudal distance from Bregma. Single arrows in green indicate the LC horns, double arrows in green indicate the LC central core, and blue arrows indicate mesencephalic trigeminal nucleus (MeV). *B*: a reproduction of Loughlin’s depiction of the LC from a lateral view showing the central core and horns (67). *C*: two atlases of the rat brain differ in their depiction of the rostral and caudal aspects of the LC. Paxinos and Watson’s atlas shows a caudal horn but no rostral horn, whereas Swanson’s atlas shows a rostral horn but no caudal horn. The three-dimensional (3-D) renderings were constructed from coronal sections of each atlas for comparison using the neuroVIISAS platform (68, 69).

Even in species with a well-defined LC core, confirmation of cells as LC neurons can be confounded by confusion over the anatomical borders of the LC. The core of LC has a well-characterized cytoarchitecture (11, 53, 54, 66). Its cytoarchitectonic features include a dense packing of cell bodies in the gray matter surrounding the fourth ventricle (the so-called periventricular gray) and a lens-shaped grouping of cells elongated in the dorsoventral axis (when viewed in the coronal plane). At the rostral and caudal ends of the nucleus, the LC core contracts to form tubular extensions of compacted cells extending from both rostral and caudal ends of the nucleus into the periventricular gray. Thus, within the brainstem, the most rostral and caudal aspects of the LC appear circular rather than oval-shaped in the coronal plane (11, 67). These rostral and caudal narrowings of the core can be termed the rostral and caudal horns of the LC core (Fig. 3, *A* and *B*). It is critical to note that only the caudal horn of the LC core is depicted in Paxinos and Watson’s Rat Brain Atlas (70), whereas only the rostral horn of the LC core is depicted in Swanson’s Rat Brain Atlas (71) (Fig. 3*C*). This discrepancy is due to the fact that, in both atlases, the coronal sections were too thick to accurately parse the rostro-caudal structural changes of the LC. By chance, the sectioning for one atlas captured the rostral horn, whereas the sections for the other atlas happened to capture the caudal horn. Neither atlas captures the LC in its entirety. Based upon existing studies, the forebrain projection targets of the rostral and caudal horns of the LC core differ from one another and from the central LC core (72). The physiology of LC horn neurons has, to our knowledge, not been characterized and compared with the central portion of the LC core in any species. A final aspect of the LC core cytoarchitecture is that, when viewed in the sagittal plane, many of the neurons are “lens shaped…with the perpendicular long axes oriented anteroposteriorly…” (11). In rodents, three noradrenergic cell morphologies have been observed: multipolar, fusiform (lens shaped), and round (67, 73–75). To date, there has been no physiological differentiation of these cell types.

**Figure 4.**
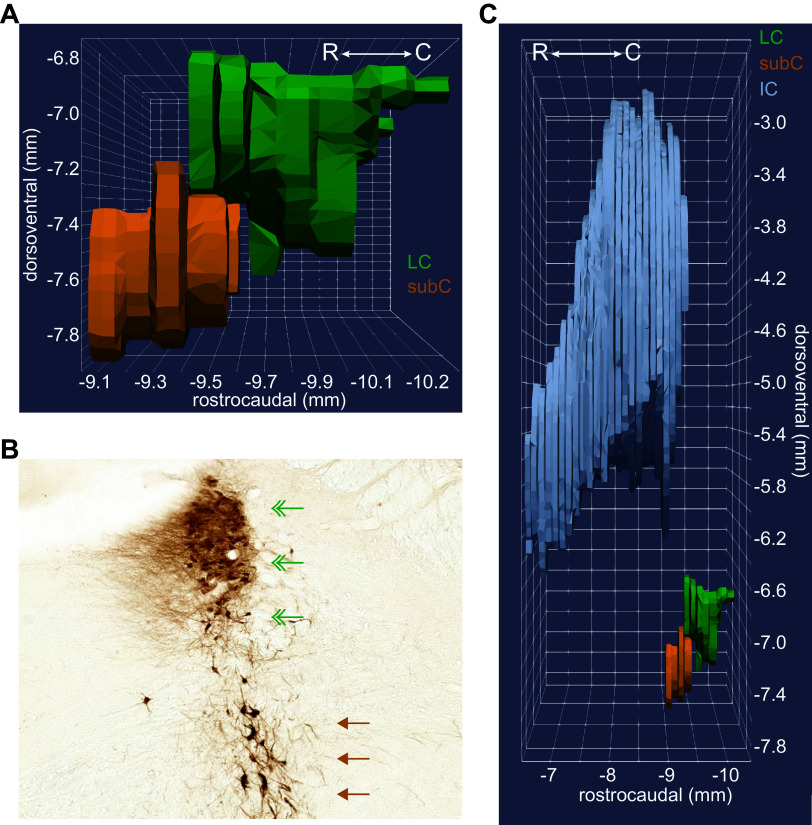
The subcoeruleus (SubC) is ventral to the rostral aspect of the locus coeruleus (LC) core and extends far rostral into coronal sections containing the inferior colliculus (IC). *A*: the three-dimensional (3-D) rendering shows the SubC region (orange) ventral to the LC core (green). The rendering was constructed from coronal sections in Paxinos and Watson’s rat brain atlas (70), with permission from Elsevier. *B*: a coronal section from the rat brain at the level of the rostral horn. Norepinephrine (NE) neurons appear as brown using a diaminobenzidine (DAB) and horse radish peroxidase reaction to visualize a tyrosine hydroxylase antibody. The rostral horn of the LC core is indicated by green double arrowheads. The SubC is indicated by brown single arrowheads. Note that SubC neurons are larger and arranged more diffusely compared with the LC core. This coronal section is from approximately −9.5 mm to −9.4 mm in the rostrocaudal plane, as shown in *A*. Note that the 3-D rendering in *A* does not appear to be a “horn” because that aspect of the LC is not contained within Paxinos and Watson’s rat brain atlas. *C*: the 3-D rendering shows the SubC extending rostrally into sections also containing the caudal aspects of the IC (gray), whereas LC neurons are not present in coronal sections containing the IC. Thus, the IC can be used in sagittal and coronal sections to determine whether NE neurons ventral to the 4th ventricle are LC core or SubC.

**Figure 5.**
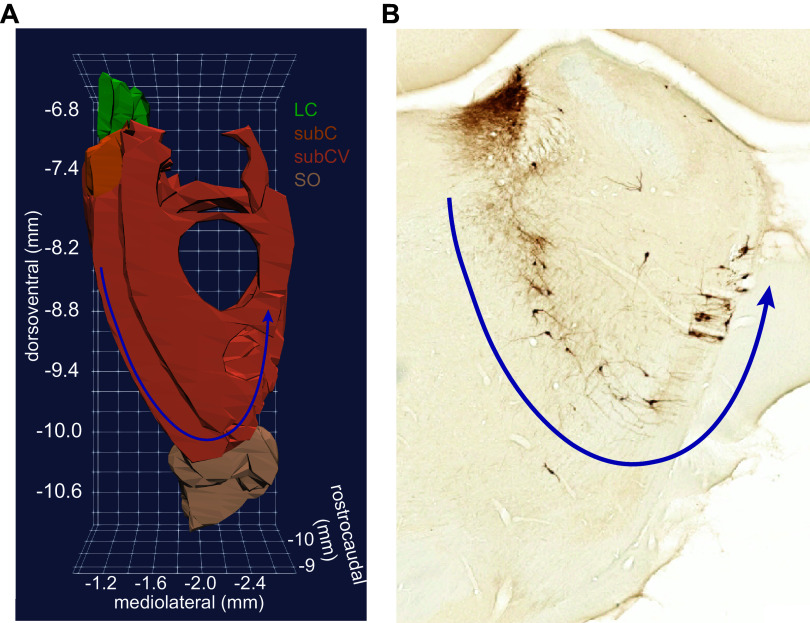
Ventral aspect of the subcoeruleus (SubCV) neurons are diffusely distributed in the ventral pons. *A*: the three-dimensional (3-D) rendering of coronal sections from Paxinos and Watson’s rat brain atlas [Paxinos and Watson (70), with permission from Elsevier] shows the SubCV area extending ventrally until the superior olive (SO). The view is from the rostral aspect of the brain, looking in the caudal direction through the SubCV, followed by the subcoeruleus (SubC), and then the locus coeruleus (LC). The norepinephrine (NE) neurons of the SubCV are arranged in an arc around the trigeminal nerve, which appears as a “hole” in the 3-D rendering. The arc is indicated by the blue arrow. *B*: a coronal section from the rat brain at the level of the rostral horn of the LC core. NE neurons appear brown using a diaminobenzidine (DAB) and horse radish peroxidase reaction to visualize a tyrosine hydroxylase antibody. The arc of SubCV neurons around the trigeminal nerve is indicated by the blue arrow. Note that the SubCV neurons are sparse and scattered in a highly diffuse arrangement.

**Figure 6.**
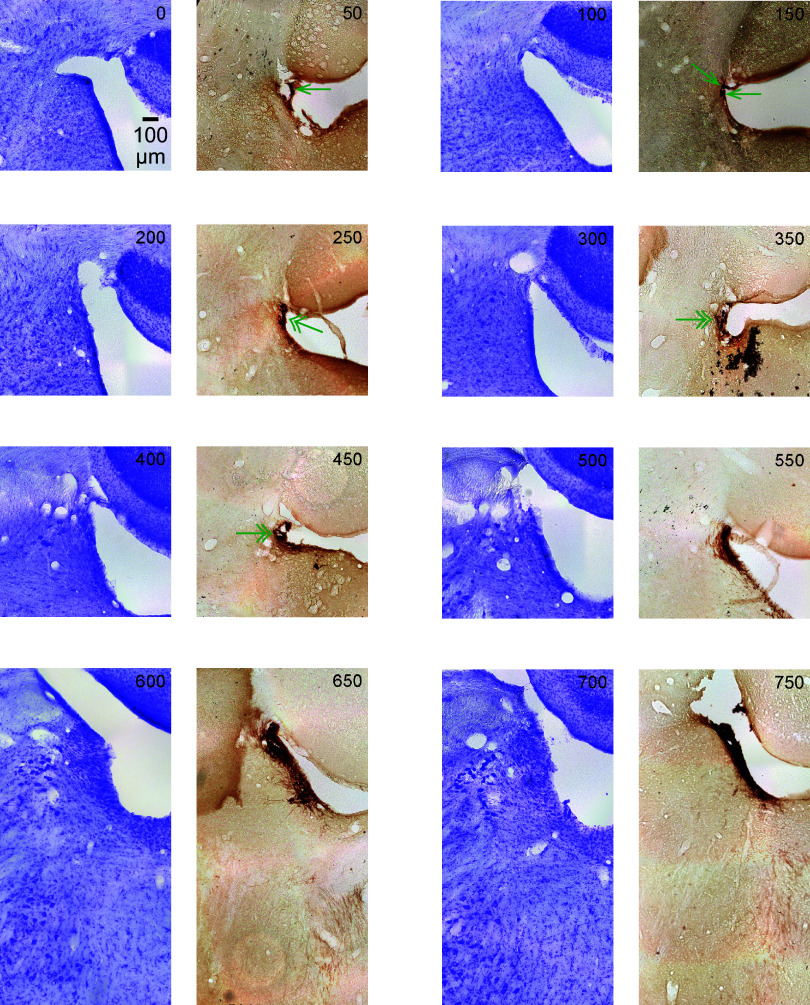
The figure shows serial coronal sections of the rat (male) brainstem in 50-μm thickness. All sections are the same scale (see 100 μm scale bar). Upward is dorsal and leftward is lateral. The sections alternate Nissl (violet) and tyrosine hydroxylase (TH) immunostain (dark brown). This figure contains the most caudal sections, relative to Figs. 7, 8, and 9. The *top left* section is the most caudal section and is labeled 0. Subsequent serial sections are labeled in 50-μm increments from the first section and continue rostral (through Figs. 7, 8, and 9). In the first few caudal sections, where locus coeruleus (LC)-norepinephrine (NE) neurons are sparse, a single green arrowhead marks TH-positive neurons. In the next few sections, areas of increased density of TH-positive neurons are shown with double arrowheads. In subsequent sections, the TH-positive neurons are densely stained and are therefore left unmarked.

**Figure 7.**
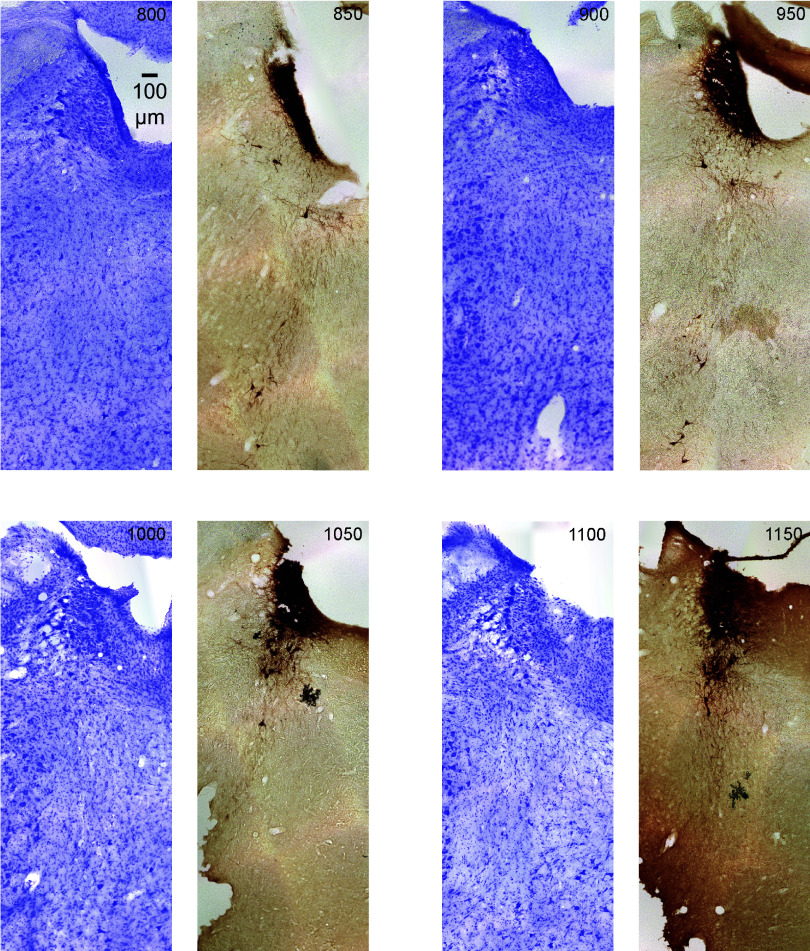
The figure shows serial coronal sections of the rat (male) brainstem in 50-μm thickness continuing from Fig. 6. All sections are the same scale (see 100 μm scale bar). Upward is dorsal and leftward is lateral. The sections alternate Nissl (violet) and tyrosine hydroxylase (TH) immunostain (dark brown). The *top left* section is labeled in μm from the most caudal section in the *top left* corner of Fig. 6. Subsequent serial sections are labeled in 50-μm increments and continue rostral (through Figs. 8 and 9).

**Figure 8.**
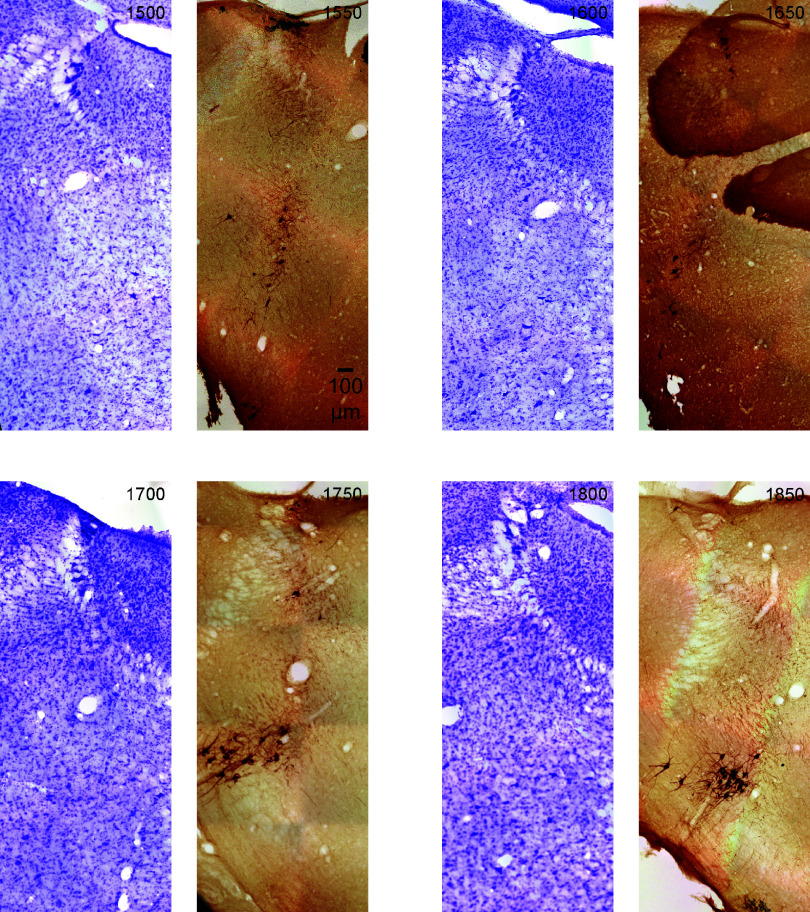
The figure shows sections that are rostral to the locus coeruleus (LC) central core. The final section of the LC rostral horn is shown in the section marked 1250. Subsequent sections are beyond the boundary of the LC. Note that these sections still, however, contain tyrosine hydroxylase (TH)-positive neurons, such as subcoeruleus (SubC)-norepinephrine (NE) neurons. The immunostained (dark brown) neurons are in the dorsal brainstem (SubC). The images are serial coronal sections of the rat (male) brainstem in 50-μm thickness continuing from Figs. 6 and 7. All sections are the same scale (see 100 μm scale bar). Upward is dorsal and leftward is lateral. The sections alternate Nissl (violet) and TH immunostain (dark brown). The *top left* section is labeled in μm from the most caudal section in the upper left corner of Fig. 6. Subsequent serial sections are labeled in 50-μm increments and continue rostral (through Fig. 9).

**Figure 9.**
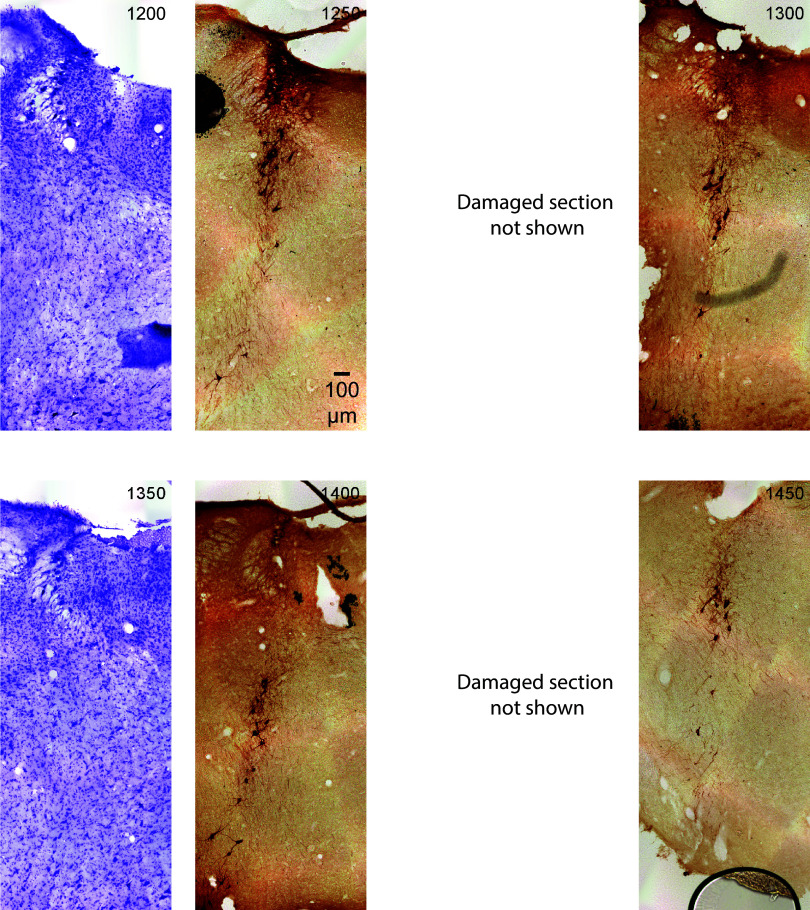
The figure shows sections that are rostral to the locus coeruleus (LC) rostral horn (see Fig. 8. section marked 1250). Therefore, these sections run 250 μm to 600 μm rostral to the most rostral boundary of the LC. However, these sections still contain tyrosine hydroxylase (TH)-positive neurons, such as subcoeruleus (SubC)-norepinephrine (NE) neurons. Note that the section at 1650 contains an artifact, which is cerebellum tissue laying over the brainstem. The immunostained (dark brown) neurons are in the dorsal brainstem (SubC). The images are serial coronal sections of the rat (male) brainstem in 50-μm thickness continuing from Figs. 6, 7, and 8. All sections are the same scale (see 100 μm scale bar). Upward is dorsal and leftward is lateral. The sections alternate Nissl (violet) and TH immunostain (dark brown). The *top left* section is labeled in μm from the most caudal section in the upper left corner of Fig. 6. Subsequent serial sections are labeled in 50-μm increments and continue rostral (through to the *bottom right* of Fig. 9).

A group of NE neurons immediately adjacent to the LC core, and potentially confused with LC core neurons, is the subcoeruleus (SubC). Subcoeruleus (SubC) neurons are scattered and lie ventral to the LC core. In the rostrocaudal plane, the most caudal SubC neurons appear at the rostral aspects of the central portion of the LC core (Fig. 4, *A* and *B*). Advancing rostrally, the SubC continues ventral to the rostral horn of the LC and reaches as far rostral as the inferior colliculus (IC), which is well beyond the cytoarchitectonic boundary of the LC (Fig. 4*C*). In mice, tyrosine hydroxylase-positive neurons appear 160 μm rostral to the LC core (76). In coronal sections of rat brain that are stained for tyrosine hydroxylase (TH) or dopamine β-hydroxylase (DBH), there is a 200–400 μm region between the LC rostral horn and the SubC that is largely devoid of NE-producing neurons (see structural gap in Fig. 4*B*). This region of non-NE producing cells makes the NE-containing neurons of the rostral horn LC clearly distinguishable from the SubC. Note that this space is not apparent in the three-dimensional (3-D) rendering in Fig. 4 because the horn does not appear as narrow in the dorsoventral axis of Paxinos and Waston’s atlas, as shown in Fig. 3*C*. Additional TH-positive neurons are distributed in the deeply ventral pons, in an arc around the trigeminal nucleus, until just dorsal to the superior olive (Fig. 5). The trigeminal nucleus appears as a “hole” in 3-D rendering shown in Fig. 5*A*. These more ventral NE neurons are termed the ventral aspect of the subcoeruleus (SubCV). The SubCV has also been termed the A7 adrenergic cell group (77). Figures 6, 7, 8, and 9 illustrate the juxtaposition of the LC, SubC, and SubCV. These figures show serial, 50-μm thick coronal sections (alternating Nissl and TH immunostaining) of the rat (male) brainstem from the most caudal LC-NE neurons (Fig. 6) until rostral levels beyond the LC (Figs. 8 and 9).

Given the proximity of the LC, SubC, and the most dorsal neurons of the SubCV, it is apparent that extracellular recordings from opto-tagged NE neurons in the dorsal pons, and even directly visualized NE neurons in GCaMP recordings, can mix populations of NE neurons in the SubC/SubCV with those of the rostral aspect of the LC core (including both the central core and its rostral horn). For instance, some of the more rostral NE neurons shown in a recent LC study might be SubC neurons (78). Consistent with the LC defined across six 100-μm coronal sections in the Allen mouse brain atlas, it is apparent from Fig. 1, A and *C* in Ref. 78 that the mouse LC core (labeled −300 μm to +200 μm) is a compact structure surrounded by GAD+ interneurons that have little-to-no intermingling within the LC core. Note that other work in mice has also shown little-to-no intermingling (79). However, in the more rostral slices (labeled +300 and +400) from the study by Breton-Provencher and Sur (78), the NE-positive neurons are ventrally displaced, consistent with a location in the SubC. At this rostral level, there is more intermingling of GABA interneurons with the NE-positive neurons. This is consistent with rat brain anatomy, which has shown GABA interneurons in this region rostroventral to the LC (80, 81). These GABA interneurons may constitute a caudal extension of the laterodorsal tegmental nucleus (LDTg) intermingling with the SubC.

Recordings that group NE neurons of the SubC/SubCV and LC together are problematic because little is known about the functional, behaviorally relevant physiological responses, and the anatomical connections of SubC/SubCV neurons in even the most commonly used species (mice, rats, and nonhuman primates), as well as in nonmammalian species. However, histological examination of the location of the recording electrode or plane of imaging can determine whether an NE neuron should be included in the LC or SubC/SubCV because of cytoarchitectonic differences between these structures in rats and mice, other mammals, and in avians (11, 55, 60, 66, 76, 82, 83). First, compared with LC neurons, SubC/SubCV neurons are noticeably larger. Their location is bisected by the fifth mesencephalic tract placing them in the parvicellular reticular nucleus instead of the periventricular gray, where the LC core is located (55). Second, SubC neurons are more scattered than LC core neurons and SubCV neurons are even more diffusely distributed. In sagittal or coronal sections, ventrally displaced NE-positive cells observed at the level of the inferior colliculus are part of the SubC/SubCV (Fig. 4*C*). A cautionary note: using the IC as a marker for SubC/SubCV NE neurons requires that the tissue cutting plane is level.

**Figure 10.**
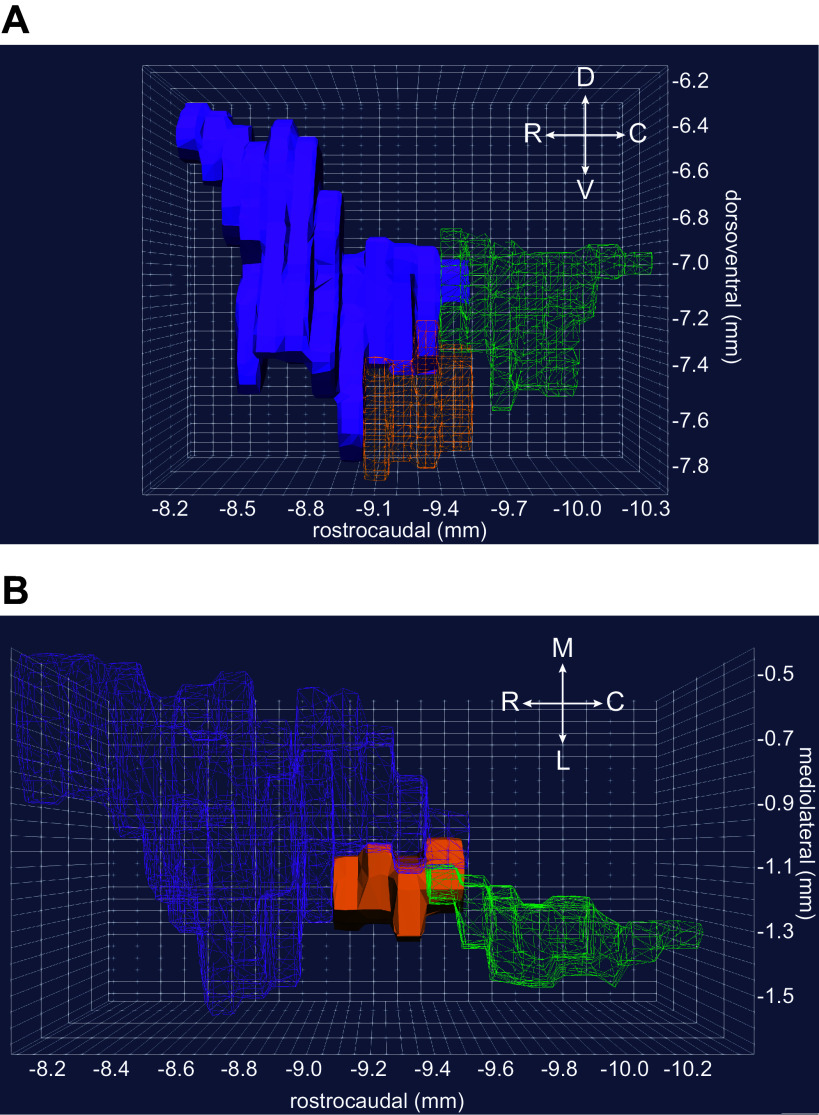
The intermingling of the rostral locus coeruleus (LC) core, subcoeruleus (SubC), and laterodorsal tegmental nucleus (LDTg) presents potential for misclassifying GABAergic, glutamatergic, and cholinergic neurons as LC core neurons. *A*: the three-dimensional (3-D) rendering shows a lateral view of the LDTg (purple), SubC (orange), and LC (green). The SubC and LC are shown as wireframe renderings to highlight the overlap of the LTDg with norepinephrine (NE) neurons of the SubC and LC. In a sagittal section, the GABA, glutamate, and acetylcholine producing neurons of the LDTg can appear intermixed with NE neurons of the LC core. *B*: the 3-D rendering shows a dorsal view, looking ventrally into the pons. This view illustrates the close proximity of rostral LC core (green) and LDTg (purple). The LDTg and LC core are presented as wireframe renderings to emphasize that inserting a multi-electrode array near the rostral LC core with a slight angle can easily penetrate the LDTg, LC core, and SubC (orange) in a single-recording tract, or simultaneously record from LDTg on dorsal aspects of the array and SubC on ventral aspects of the array while missing the LC core altogether. This complication can be avoided when recording tracts are confined to the more caudal, central portion of the LC core (e.g., −9.7 to −10.1 mm on the rostrocaudal axis). In both panels, the 3-D renderings were constructed using coronal sections from Paxinos & Watson’s rat brain atlas [Paxinos and Watson (70), with permission from Elsevier].

NE neurons in the rostral horn of the LC core, as well as those in the SubC intermingle with GABA interneurons, glutamate neurons, and cholinergic neurons contained in the caudal ending of the laterodorsal tegmental nucleus (LDTg) (84, 85). Therefore, GABAergic, glutmatergic, and cholinergic neurons can be improperly included in recordings of the rostral horn of the LC core (Fig. 10). It is particularly important to note that GABA interneurons are not present within the LC core of rats and mice (33, 79, 80, 86). A population of fast-spiking GABA interneurons does lie ∼300–500 µm beyond the ventromedial border of LC core in rats (80) and ∼200–400 µm beyond the border of the LC core in mice (79) in an area termed the rostroventral pericoerulear region (81) (At present, this anatomical region has not been included in Paxinos and Watson’s or Swanson’s rat brain atlases). These interneurons project nearly exclusively in the region immediately surrounding the LC core, where dendrites of LC-NE cells project and ramify (79, 81). Thus, the notion of an inhibitory GABAergic “shell” projecting solely to the LC core may be appropriate and anatomically similar to the relationship between the thalamic reticular nucleus and the core thalamic nuclei. However, such interneurons are not part of the LC core. Given that GABA interneurons are scattered throughout the brainstem, identifying the peri-LC inhibitory shell neurons requires viral tracing (79).

In sum, in the mouse and rat brain, SubC NE cells are scattered (not tightly packed as in the LC core) and are located well beyond the cytoarchitectonic boundaries of the LC core where they intermingle with GABA interneurons. Are SubC neurons merely displaced from the LC core as a result of developmental pressures? Should the definition of the LC be extended—beyond the core—to include the scattered SubC-NE neurons and the intermingled GABA interneurons? Should peri-LC GABAergic neurons be delineated as an LC “shell” region? Answers to these questions currently elude the field because the functional, behaviorally relevant physiological response, and the anatomical connections of SubC NE and peri-LC GABA neurons are relatively unexplored in mice, rats, and nonhuman primates and not characterized in other species. It is worth noting that the dorsal SubC neurons share a developmental origin with the LC core, but SubCV neurons do not (87). However, defining the LC to include SubC neurons may encroach upon the LDTg leading to inclusion of other cell types, such as GABA interneurons, which are not part of the cytoarchitectonically defined LC core. In the absence of experiments characterizing the features of SubC neurons and peri-LC neurons, we suggest that NE and GABA neurons outside of the cytoarchitectonically defined LC core in rats and mice be labeled as SubC neurons and rostroventral peri-LC neurons, respectively, rather than as “LC-NE” or “LC-GABA” neurons. Since it is possible to include LC core-adjacent NE and GABA-expressing neurons in recordings of the LC core, we provide consensus guidelines to help define whether extracellularly recorded neurons are within the core of the nucleus. These guidelines apply to species such as mice and rats or any other species with a core group of NE-producing LC neurons that can be defined using cytoarchitectonics (55). Although these guidelines are benchmarked to mice, rats, and nonhuman primates, they can be applied to other species by modifying them to account for what is known about the physiology of LC-NE neurons and the cytoarchitecture of the LC in that species. When less is known, performing all of the tests in these guidelines will inform the field about key similarities across species, and drive investigation of any differences that emerge. Especially when less is known, it is prudent to exercise caution regarding cellular identity based solely upon extracellular recordings instead of through multiple means.

Definitive identification of LC neurons in extracellular recordings has always been technically demanding. The challenge increases as discoveries are made that broaden the spectrum of electrophysiological, genetic, neurochemical, and projection target diversity in LC neurons, both within and across species (5). Our continued progress in understanding LC function across species will be hastened by thorough characterization of recorded neurons. Here, we highlighted the use of multiple methods in combination to ensure that recorded neurons are LC-NE core neurons, which include four key physiological criteria, the use of linear probes that take into account the anatomy of the LC core, and histology with appropriate section thickness and immunohistochemistry to enable detailed reporting of electrode location with respect to consensus anatomical boundaries of the LC core. Although greater confidence in the identity of recorded neurons may also be achieved using GCaMP imaging of NE-containing neurons, the potential for confounding LC core and SubC NE neurons remains, in the absence of detailed histological verification. In addition, the biphasic response of LC core neurons and LC neuronal ensemble activity patterns will be inaccessible with the GCaMP indicators that have been used in the LC. However, observing the biphasic response of LC neurons may be permissible with newer versions of GcaMP as long as the baseline fluorescence is high enough to observe inhibition of neurons with an ∼1-Hz spike rate (88). Additional features of LC neurons that will provide new insights into LC-NE function across species are the receptor complement of individual cells, cotransmitter expression, and the developmental origins of various cell types. Overall, the expectation is that comparative neurobiology approaches, in combination with multiple methods for recording LC neurons, will greatly advance our understanding of the LC from finches to fish and beyond.

## CONSENSUS GUIDELINES FOR LC EXTRACELLULAR RECORDINGS ACROSS SPECIES

### Guideline 1: Test and Report Four Key Physiological Criteria for Identifying LC Cells

These are *1*) 2–3 ms duration of the entire waveform, *2*) mean spontaneous firing rate of the recorded population is ∼1 Hz and no higher than 2 Hz (except in specific states in which the population mean may be as high as 3 Hz), *3*) a biphasic response to a brief sensory stimulus (of appropriate modality given the state of the organism), and *4*) clonidine-induced inhibition of single unit and multiunit activity. Note that the highpass filter cutoff affects waveform duration, so comparisons of waveform can only be made across studies using the same highpass filter and identical measure of waveform duration (e.g., entire waveform or a peak-trough latency). Depending on these factors, the LC neuronal waveform may be less than 2–3 ms.

### Guideline 2: Advance the Electrode through the Dorsoventral Axis of the Brainstem While Monitoring Activity to Assess Whether the Electrode Is in the Intended Target (Central Portion of the LC Core or in the Rostral or Caudal Horn)

The depth span over which signs of LC-NE neuronal activity are observed provides evidence for determining electrode placement in the central core or one of the horns. When lowering an electrode ventrally from the most dorsal point at which the electrode first detects LC activity, if the stimulus-evoked biphasic response disappears after 200 µm (in rat), then the recording is in either the rostral or caudal horn portion of the LC core. Note that this method cannot distinguish the rostral horn from the caudal horn. When recording in the LC core (in rats), at 500 µm depth from the most dorsal aspect, the electrode should move out of the LC and the biphasic response should disappear. These track distances for rats are based on Nissl-stained coronal sections. They must be adapted for use in other species based on the brain atlas of those species.

### Guideline 3: Use Precise Histological Identification of Recording Electrode Placement

Reporting histology with consistent anatomical terminology across studies is critical for identifying features of LC neuronal activity across species and distinguishing potential differences between species.

### Guideline 4: Use Maximally 50-µm Coronal Section Thickness in Rats (or 25 µm in Mice) and Include, Alongside DBH Immunohistochemistry, Choline Acetyltransferase Immunohistochemistry Marking Cholinergic Neurons to Assess Whether the Recorded NE Neurons Are Encroaching on the LDTg

We recommend these section sizes to sample frequently enough to track the transition between the horn portions and central portion of the LC core. We encourage researchers working in any species to obtain coronal sections at the level of the IC (rostral to the LC core) and report whether the electrode was recording from SubC-NE neurons, which continue rostral to the LC, until the level of the IC.

## GRANTS

This work was supported by the Research Council of Finland PROFI6 funding program (UHBRAIN project), start-up funding from the Helsinki Institute of Life Science at the University of Helsinki, and by National Institutes of Health BRAIN Initiative Grant 1R34NS123876.

## DISCLOSURES

A.E.P. has grant funding from Eli Lilly and has provided consultancy to Eli Lilly and Lateral Pharma for projects unrelated to this topic. None of the other authors has any conflicts of interest, financial or otherwise, to disclose.

## AUTHOR CONTRIBUTIONS

N.K.T. conceived and designed research; A.V. analyzed data; A.V. and N.K.T. prepared figures; N.K.T. drafted manuscript; A.V., G.A.-J., A.E.P., G.R.P., B.W., and N.K.T. edited and revised manuscript; A.V., G.A.-J., A.E.P., G.R.P., B.W., and N.K.T. approved final version of manuscript.
